# Dr. Edwin A. Stebbins

**Published:** 1896-11

**Authors:** 


### Obituary.

### DR. EDWIN A. STEBBINS.

Dr. Edwin A. Stebbins died at Shelburne Falls, Mass., on September 26, 1896. He had been ill with an incurable disease for over a year. Dr. Stebbins was born in Brookline, Windham County, Vt., July 10, 1837. He was reared on a farm and educated in the public as well as other schools, and became a teacher subsequently of ornamental penmanship in the Wesleyan Academy, Springfield, Vt.

He began the study of dentistry at Benson, Vt., and opened his first dental office at South Londonderry, Vt., in 1860. In 1862 he enlisted in Company G, Eleventh Regiment, Vermont Volunteers, and served until the close of the war. He was commissioned first lieutenant after having spent some time as quartermaster-sergeant. In the spring of 1864 he was ordered to the front and placed in the Sixth Corps. The regiment was with Sheridan in his expedition up the Shenandoah Valley, was in front of Petersburg in 1864–65, and took part in the final assault upon Lee.

At the close of the war he resumed practice first at West Townshend, Vt., and finally settled at Shelburne Falls in 1869.

Dr. Stebbins came prominently before the dental profession through his work with nitrate of silver upon children's teeth. This had been so thoroughly and so carefully performed that it at once attracted wide attention, and, although some efforts were made to minimize this in certain quarters, they were not successful, and Dr. Stebbins was given due credit for original and successful investigations.

It was through this effort that the writer became first acquainted with him, and learned to appreciate his character as one of the most painstaking and earnest men in the profession, but at the same time entirely free from self-assertion. Those who were in attendance at the American Dental Association, at Saratoga, where, upon motion of the writer of this, Dr. Stebbins was granted the special privilege of detailing this process, will recall the deep impression his careful statements and scientific method made upon the interested auditors. It is not remarkable, therefore, that his experiments with nitrate of silver, in the direction described, were subsequently amply confirmed in practice.

He was an active worker in the church and Sunday-schools, and was also an earnest temperance advocate, taking an active interest in the Prohibition Party in his State. He was also interested in Masonry.

He was active in school work and served for many years as one of the trustees of Arms' Academy.

In the death of Dr. Stebbins the profession of dentistry has lost, in the language of one of his colleagues, "one of its truest members." The silent reaper spares neither high nor low, but while the departure of a noble man from the ranks must be a cause of painful regret, the consolation remains, to his family and his profession, that his work in life stands as the truest and best monument to his honor.

